# Prominent Causes of Failure in the Use of Electricity in Dentistry

**Published:** 1898-02

**Authors:** C. S. Neiswangey

**Affiliations:** Chicago.


ARTICLE IX.

### PROMINENT CAUSES OF FAILURE IN THE USE OF ELECTRICITY IN DENTISTRY.

BY C. S. NEISWANGEY, M. D., CHICAGO.

Read before Southern Dental Association, Old Point Comfort, August, 1897.

Electricity is a natural phenomenon, and the only way to accomplish results, either physical or therapeutical, is to observe closely and try to interpret the language in which nature speaks to us. Electricity is closely related with some other forces with which we think we are familiar. It is but a mode of motion or under manifestation of a form of matter called the ether, which permeates all bodies and pervades all known space.

Light is only a transverse vibration of this same ether, with infinitesimally short wave lengths. Heat and sound are made apparent to us by the aid of longer waves of the same medium. Electricity is governed by laws which are as well known as those which regulate light, heat, sound and gravitation, all elucidated by mathematical process. All animal and vegetable life being due to and dependent upon the electrical conditions surrounding it, is it not reasonable to suppose we should be able to restore the equilibrium (health) of the body by supplying electricity from an outside source?

To comprehend the subject of electricity there are three fundamental principles which must be observed: First. *Voltage.* This is not electrity itself but the force which impels it—push-power—produced by a difference of electric level. Second. This voltage or difference of electric level is caused (if we are doing cataphoric work with a battery of cells) by the difference of potential between the two elements within the cells. If two pieces of metal are immersed in an acid or other existing fluid, one of which is acted upon by the acid much more readily than the other, the voltage will be in proportion to the difference with which two metals are acted upon by the fluid. Two metals having a great difference of potential will give a high voltage or push-power. Third. Amperage is electricity or current; it must not be confounded with voltage. We may represent this by a rain; the water represents the amperage, the swiftness with which it flows the voltage; hence as we may have a broad river flowing slowly, or a narrow stream running swiftly, so we may have high amperage and low voltage, or vice versa.

There are three factors in the causes of failure in this class of work: (1) The battery. (2) The controlling device. (3) The manipulation of the electrodes.

(1) The galvanic current—the direct or continuous current generally utilized in dentistry—is produced by chemical decomposition; the conversion of chemical energy into electric energy. If we would generate electric energy sufficient for our purpose, we must have a corresponding consumption of the fuel that keeps up the chemical decomposition, and this fuel must be renewed as required. Hence the failures of those who are seduced by the attractive advertisements of those who furnish a battery of a few cells as large as a man's finger guaranteed to do heavy work for years. In dentistry we have a heavy resistance to the passage of the current to overcome, and as it is the current or amperage that does the work, we must have sufficient voltage or push-power in our battery to overcome this resistance.

(2) The controlling device should be capable of controlling the current in absolutely gradual gradations, and not by steps, or cell by cell, as this would cause very great pain in a hypersensitive cavity. An instrument in which the resistance is composed of german silver will, for instance, be objectionable, because the passage of electricity generates heat, and when the wire is heated resistance is increased and the current drops back and has to be re-established by a forward movement of the instrument. This is a very prominent cause of failure not heretofore recognized. With the wire rheostat there is a constant fluctuation of the current backwards and forwards.

The term "volt selector" means nothing. A volt selector is a current controller, and the more voltage or pressure you have the more current is pushed through the resistance we encounter. It is the amount of current that does the work, and a milliamperemeter is the proper thing to have in the circuit that we may know how much current is passing through. Although the amount of current necessary in any certain case has never been definitely determined, it is a satisfaction to see the indicator upon the meter move and to know that the patient gets just what is indicated upod its face.

Let the battery have at least a pressure of forty or sixty volts. Less may do some of the work, but you have a reverse force for other cases. Your current controller should increase and decrease the resistance gradually and not by steps. It should be of graphite or carbon with the patient in shunt. The electrode should be supported and controlled by the hand. A good meter with range of scale to five or more milliamperes, graduated in one-tenth milliampere divisions, should be in the circuit. With such apparatus, and by paying due regard to polarity and the electro-sensibility of your patient, you will have no reason to regret the use of this valuable agent. If you have not the time to devote to it do not attempt its use. If you are not overburdened with practice there is no better way to increase it than by its judicious use.

### DISCUSSION.

Dr. J. Rollo Knapp asked Prof. Neiswanger to explain why, in view of the decomposition and waste of the two metals used in the battery cells, there was not a corresponding waste in the two metals used as fillings in a tooth. Is the loss so very gradual that it is imperceptible? Dr. Neiswanger replied that it was probably because the two metals were not kept submerged.

Dr. Marshall said that Dr. S. B. Palmer had established the fact years ago, that there is a current between the two metals in a tooth until the surface is oxidized, as seen in the blackened surface of an amalgam filling.

Prof. Neiswanger spoke of the importance of knowing which pole to use in applying medicaments. If potassium iodid is used at the negative pole the iodin would act as an acid and seek the positive pole, the potassium remaining at the negative. On the other hand, if the potassium iodid was applied on the positive pole, the iodid would remain at the positive, and potassium, the metallic base, be carried to the negative and left in the tissues. If a copper needle is used for the positive pole the copper would be decomposed by the action of oxygen, the copper going to the other pole; and in this way it would almost equal bichlorid of mercury, besides being non-toxic. Solution or compounds are always decomposed, the base remaining at the negative, provided there is sufficient current to effect the decomposition. With cotton and the alternating current you can produce a local anesthesia of the teeth, tetanizing the nerve through fatigue of the muscle.

Dr. John Marshall said that he had valuable results in that line, in relieving pain and reducing inflammation and congestions. No medicament is required, the current itself contracting the muscles of the blood vessels and producing anemia of the parts.

Dr. price spoke of the various theories as to the action in cataphoresis, and said: When all the various theories have been analyzed, we are forced to the conclusion that it is electrolysis, pure and simple, with perhaps some osmosis. —*Ohio Dental Journal.*

